# New Remedy for Strangury

**Published:** 1846-08

**Authors:** 


The following new remedy will not strike the reader as a very prepossessing addition to the materia Medica. We have been informed, however, by a practitioner of this city, that in a case of strangury in which the introduction of the catheter was attempted without success, the prescription was tried with immediate relief; and it was subsequently repeated daily, with the same results, until the occasion for its administration ceased. The cook's oracular direction in roasting a hare is, first to catch the hare; this preliminary is more emphatically applicable in the present instance! Provided the bees can be caught, and the patient's mind reconciled to the "tea," we think the prescription worthy of trial. In how much the effect is due to the idea of taking so singular a remedy, we leave our readers to judge.—*Editor Buffalo Journal.*

*New Remedy for Strangury.*—Dr. F. Gordon, of Wilson Co., Tenn., in an article on Epidemic Metritis, (*West Journ. Med. and Surg.*, Nov. 1845.) gives the following account of a new remedy, which, he says, was successful in the treatment of strangury which was a common attendant on the disease. This remedy was an infusion of the apis melifica, or honey bee. "Sweep 40 to 60 bees into a pan of water, so at to make them manageable; put the whole into a teacup, pour one gill of boiling water on them, and cover the cup securely. When it has remained twenty minutes, pour off the infusion and let the patient take the whole at a draught. This remedy relieved the strangury in from two to fifteen minutes with great certainty.

"It was introduced into the practice of medicine some years ago by Mrs. Perry, an old woman in the habitual practice of midwifery in the county of Smith; and experience proves that its efficacy is not less considerable because of its unscientific origin. Some six or seven practitioners in that section of country (some of whom do honour to the profession,) have given the remedy numerous fair trials, and, so far as I have learned, all estimate it highly. The writer has tried it repeatedly in the retention of urine from inflammation of the bladder, and from the effects of cantharides, and found it to be more prompt and certain than any other remedy. There can be no question but that the 'bee tea' will prove a valuable accession to our materia medica. How far it may be found to be useful in ischuria and disuria from every variety of cause, remains to be tested ; and its value affords abundant encouragement for further investigation.

"As to the class to which this agent naturally belongs, I have but little hesitation in placing it among the *narcotics*, That it acts as an antipasmodic there can be no doubt, but whether it is a specific for the sphincter muscle of the urinary bladder, as the ergot is supposed to be a specific for certain fibres of the uterus, has not been clearly determined.

"It is probable that the virus ejected by the bee in poisoning the wound i- inflicts upon its enemy, is the material which gives virtue to the bee tea, This virus is secreted and collected in a sac in the abdomen of the bee, near the base of its lance or sting. When war is made upon its enemy, the virus is injected into the puncture made by the lance, and the wound is poisoned. It is also emitted during the anger of the insect, as is known by its peculiar pungent odor; and that the virus which gives out this odor is the same which imparts the antispasmodic virtue to the infusion, is evinced by two facts.

"1st. The tea, when recently made, has a taste and smell identical with the odour of the incensed bee, and now the infusion is efficacious.

"2d. But if the infusion be allowed to stand and cool, and especially if allowed to remain uncovered, its characteristic odour, and taste disappear, and the tea is correspondingly inefficient. These facts justify another conclusion—that the virus is quite volatile, and requires care to prevent its escape.

"Whether this valuable virus may not be collected and concentrated, or combined with some chemical element, so as to render it portable and convenient, is a matter of interest and well worth the attention of the chemist."—*Am. Jour. Med. Sciences.*

